# Measuring Social Desirability in Collectivist Countries: A Psychometric Study in a Representative Sample From Kazakhstan

**DOI:** 10.3389/fpsyg.2022.822931

**Published:** 2022-04-06

**Authors:** Kaidar Nurumov, Daniel Hernández-Torrano, Ali Ait Si Mhamed, Ulzhan Ospanova

**Affiliations:** ^1^JSC Information-Analytic Center, Nur-Sultan, Kazakhstan; ^2^Graduate School of Education, Nazarbayev University, Nur-Sultan, Kazakhstan

**Keywords:** social desirability bias, Marlowe-Crowne, MCSDS, validation, Kazakhstan, collectivist culture

## Abstract

Social desirability bias (SDB) is a pervasive measurement challenge in the social sciences and survey research. More clarity is needed to understand the performance of social desirability scales in diverse groups, contexts, and cultures. The present study aims to contribute to the international literature on social desirability measurement by examining the psychometric performance of a short version of the Marlowe-Crowne Social Desirability Scale (MCSDS) in a nationally representative sample of teachers in Kazakhstan. A total of 2,461 Kazakhstani teachers completed the MCSDS – Form C in their language of choice (i.e., Russian or Kazakh). The results failed to support the theoretical unidimensionality of the original scale. Instead, the results of Random Intercept Item Factor Analysis model suggest that the scale answers depend more on the method factor rather than the substantial factor that represents SDB. In addition, an alternative explanation indicates that the scale seems better suited to measuring two SDB correlated factors: attribution and denial. Internal consistency coefficients demonstrated unsatisfactory reliability scores for the two factors. The Kazakhstani version of the MCSDS – Form C was invariant across geographic location (i.e., urban vs. rural), language (i.e., Kazakh vs. Russian), and partially across age groups. However, no measurement invariance was demonstrated for gender. Despite these limitations, the analysis of the Kazakhstani version of the MCSDS – Form C presented in this study constitutes a first step in facilitating further research and measurement of SDB in post-Soviet Kazakhstan and other collectivist countries.

## Introduction

Self-reports are an essential tool in the social sciences and the most commonly used assessment and data collection instruments in disciplines such as psychology (Robins et al., 2007), education (Falchikov and Boud, 1989), and sociology (Clair and Wasserman, 2007). The popularity of self-report measures arises from their easy interpretability and administration, the richness of information, motivation to reflect on the self, and sheer practicality (Paulhus and Vazire, 2007, p. 227). However, the self-report method has been a frequent target of criticism. One of the most vigorous controversies around self-report assessment has been concerning social desirability bias (SDB), or the widespread tendency of individuals to present themselves most favorably with respect to social values and norms (Tracey, 2016).

Social desirability bias has indeed been a concern in personality psychology and survey research since the mid-20th century. Edwards (1957) viewed social desirability as a single dimension that can describe all personality statements. Individuals who obtain high values on the continuum are regarded to have high socially desirable responses. On the contrary, individuals with low values demonstrate low levels of social desirability. From a sociological point of view, “…social desirability as a response determinant refers to the tendency of people to deny socially undesirable traits or qualities and to admit to socially desirable ones” (Phillips and Clancy, 1972, p. 923). Consequently, the presence of socially desirable responses in self-report data is problematic and may lead to spurious correlations between variables and the suppression or the artificial alteration of relationships between constructs of interest (King and Bruner, 2000; van de Mortel, 2008).

Several approaches have been proposed in the literature to prevent or reduce SDB, including forced-choice items, neutral items, randomized response techniques, the introduction of the bogus pipeline, self-administered questionnaires, and the use of proxy subjects. In addition to these, researchers have suggested other methods to detect and measure social desirability effects (Nederhof, 1985). Among them, the use of social desirability scales is the most common. Social desirability scales are included in conjunction with the targeted questionnaire(s) as indicators of discriminant validity. Ideally, the correlation between the scores of the targeted questionnaire and the social desirability measure is zero to weak, demonstrating that the variable of interest is unconfounded with social desirability (Tracey, 2016).

Multiple social desirability scales have been developed in past decades (see Paulhus, 1991). The Marlowe-Crowne Social Desirability Scale (MCSDS) (Crowne and Marlowe, 1960) is one of the most widespread scales to measure SDB around the world (Beretvas et al., 2002). It measures social desirability as “the need to obtain approval by responding in a culturally appropriate and acceptable manner” (Crowne and Marlowe, 1960, p. 353). The MCSDS consists of 33 binary items with true or false answers on culturally sanctioned and approved but improbable behaviors (e.g., I have never deliberately said something that hurt someone’s feelings). According to Crowne and Marlowe (1964), a unidimensional construct underlies the MCSDS: “need for approval.” Thus, higher scores in the MCSDS reflect higher needs for social approval and a tendency to portray yourself more positively.

The psychometric properties of the MCSDS have been widely studied in multiple contexts and cultures, predominantly in North America (Fischer and Fick, 1993; Loo and Thorpe, 2000; Barger, 2002; Loo and Loewen, 2004; Leite and Beretvas, 2005; Ventimiglia and MacDonald, 2012), although studies involving European (Sârbescu et al., 2012; Vésteinsdóttir et al., 2015) and Asian samples (e.g., Seol, 2007) are also available. The factor structure of the scale has been extensively analyzed through exploratory and confirmatory factor analysis, and a few studies have begun to implement alternative approaches such as item response theory and Rasch measurement (Seol, 2007; Vésteinsdóttir et al., 2017). Collectively, these studies provide inconclusive evidence on the dimensionality of the MCSDS. Some studies support the theoretical unidimensionality of the scale (e.g., Seol, 2007; Vésteinsdóttir et al., 2015), while other studies provide stronger evidence for a two-factor structure (e.g., Loo and Loewen, 2004; Ventimiglia and MacDonald, 2012) or alternative factorial solutions (e.g., Loo and Thorpe, 2000; Barger, 2002; Leite and Beretvas, 2005). Reliability analyses have also shown mixed results on the internal consistency of the scores, with coefficients ranging from 0.72 (Loo and Thorpe, 2000) to 0.96 (Fischer and Fick, 1993).

Several short versions of the MCSDS have been developed to avoid excessive item redundancy and length of the full scale (e.g., Strahan and Gerbasi, 1972; Reynolds, 1982; Ballard, 1992). These forms range between 10 and 20 items and result from factor analysis techniques assuming that the MCSDS full version assesses one single dimension. Internal consistency scores of the short versions are lower but comparable to those of the full version. Moreover, they have been considered suitable substitutions and, in some cases, significant improvements in fit over the full scale (Loo and Thorpe, 2000; Barger, 2002; Loo and Loewen, 2004; Sârbescu et al., 2012). The MCSDS – Form C developed by Reynolds (1982) stands out as one of the most commonly used short forms available. It comprises 13 items and demonstrates good psychometric characteristics compared to other short versions. The MCSDS – Form C internal consistency estimates range from 0.62 to 0.89 and its scores correlate strongly with the scores on the full scale (*r* = 0.91 to 0.96) (Reynolds, 1982; Ballard, 1992; Fischer and Fick, 1993; Loo and Thorpe, 2000; Barger, 2002; Loo and Loewen, 2004; Vésteinsdóttir et al., 2015). However, confirmatory factor analyses have provided conflicting results about the factorial structure of the MCSDS – Form C, with only partial support for the unidimensionality assumption (Barger, 2002; Loo and Loewen, 2004; Leite and Beretvas, 2005; Verardi et al., 2009; Vésteinsdóttir et al., 2015).

The measurement invariance of different versions of the MCSDS has been partially supported in previous studies. For example, Kurz et al. (2016) confirmed measurement invariance between genders in the context of Malaysia. However, the authors found only partial support for measurement invariance across languages in the Chinese and English versions of the MCSDS. Concern has also been raised about the cross-cultural validity of the MCSDS scales. Differences in the tendency to respond in a socially desirable manner across countries and cultural groups have been reported in several studies (e.g., Verardi et al., 2009; He et al., 2015). For example, Middleton and Jones (2000) used the full MCSDS scale in a convenience sample of Western and Eastern university students and found that Eastern participants were more likely to deny socially undesirable traits and to admit socially desirable traits compared to Western participants. Lalwani et al. (2006) tested the hypothesis that collectivist cultures tend to engage in deception and socially desirable responses more than individualistic cultures. Their findings suggested that people from both types of cultures engage in desirable responses, although in different ways. Individualism seemed to be more associated with the tendency to report inflated views of one’s skills and capabilities, while collectivism was linked to the tendency to present self-reported actions in the most positive manner.

More clarity is needed to understand the performance of social desirability scales in diverse groups, contexts, and cultures. The present study aims to contribute to the international literature on the measurement of social desirability by examining the psychometric performance of the MCSDS – Form C in a nationally representative sample of teachers in Kazakhstan. Kazakhstan provides an interesting context to explore social desirability measurement for several reasons. First, the country occupies a strategic geopolitical location in the Eurasian mass and constitutes a unique blend of Eastern and Western cultures. Kazakhstan is in fact a diverse country with more than 120 ethnic groups that have different social values and norms (The Agency on Statistics of the Republic of Kazakhstan, 2011). Second, as a former Soviet republic, Kazakhstan maintains a strong national collectivist tradition (Winter et al., 2020). This is relevant as collectivist cultures tend to demonstrate stronger and more consistent magnitudes and patterns of SDB (Bernardi, 2006; Kim and Kim, 2016). Third, measuring SDB is particularly important in societies that have experienced authoritarian regimes in the past, such as Kazakhstan. Finally, SDB is a widespread problem that affects many areas, including education. Social desirability may explain the questionable results of the latest international evaluations such as TALIS-2018 in the context of Kazakhstan, in which teachers report values well above the OECD average in some questions. For example, 82% of Kazakhstani teachers were confident in their ability to teach using ICT (OECD average of the OECD was 67%). At the same time, 30% of teachers marked ICT for teaching as the main priority of professional development (Information-Analytic Center [IAC], 2019; OECD, 2019). Having a reliable and valid tool to measure SDB could help to account for the measurement error caused by this phenomenon in Kazakhstan, Central Asia, and other collectivistic countries.

## Materials and Methods

### Description of Sample

The sample consisted of subject teachers who participated in the UNESCO Teachers’ Readiness Survey in early 2021 in Kazakhstan (Information-Analytic Center [IAC], 2021). The survey is based on the UNESCO ICT competency framework for teachers and covers areas such as teacher ICT competencies, use of ICT in teaching, awareness of the official policy on ICT use in education and professional learning (UNESCO, 2011). To ensure large-scale representativeness, the sample design consisted of an explicit stratified selection of a proportionally allocated sample from the population list of subject teachers, as well as a weighting strategy. The latter included adjustment for unknown eligibility, adjustment for non-response, post-stratification, and extreme weights trimming. In total, 2,851 subject teachers were selected for the main study with a final response rate of 86% (*n* = 2,461). The weighted sample mean age of subject teachers is 40.58 (std. error = 0.22) whereas the population mean age is 40.50. Additional information on the distribution of the raw sample responses in biographic and geographic subgroups is presented in Table 1.

**TABLE 1:** Distribution of raw sample responses in subgroups.

	*n*	%
**Gender**		
Male	470	19.0
Female	1,991	81.0
**Language**		
Kazakh	1,507	61.2
Russian	954	38.8
Geographic locality		
Rural	1,422	57.8
Urban	1,039	42.2
**Age groups**		
18–35 years	914	38.0
36–50 years	997	41.4
51–72 years	496	20.6

*Age was transformed into the categorical variable with three categories. A 51–72 years old group though smallest in terms of the number of teachers, nonetheless, includes a larger range in years than 18–35 and 36–50 groups. This is due to a skewed population distribution toward younger teachers.*

One can notice significant gender disproportion among men – 470 (19%) and women – 1991 (81%). This disproportion is expected due to the traditional overrepresentation of women in school teaching in the context of Kazakhstan. Additionally, the distributions of responses show higher proportions of Kazakh language and rural subject teachers in terms of subgroups of language and geographic location.

### Instruments

The Marlowe Crowne Social Desirability Scale (MCSDS) – Form C (Reynolds, 1982) was used to measure social desirability bias in this study. The MCSDS – Form C is a brief questionnaire comprising 13 items that represent a selection of socially desirable and undesirable behaviors (e.g., “No matter who I’m talking to, I’m always a good listener,” “There have been occasions when I took advantage of someone”). Items are dichotomously scored on a true/false scale. A score of 1 is granted if the participant responds “true” to a socially desirable item or “false” to a socially undesirable item. On the contrary, a score of 0 is provided if the participant responds “false” to a socially desirable item or “true” to a socially undesirable item. A total score can be obtained summing up the scores for all items, with higher scores representing higher SDB.

The MCSDS – Form C was translated into the two official languages of Kazakhstan (i.e., Russian and Kazakh) using a back-translation approach (Brislin, 1970). In addition to that, the Russian and Kazakh translations of the MCSDS – Form C were further assessed by the research team to ensure understandability, psychological equivalence, and the accuracy of the translations. The MCSDS – Form C was included in the UNESCO questionnaire and distributed online. Anonymity and confidentiality were ensured, no information that could identify the identities of the participants was collected.

### Procedure and Data Analysis

**FIGURE 1:**
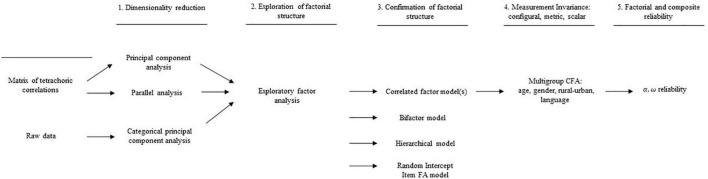
Psychometric properties of MCSDS – Form C analysis flowchart.

Descriptive analyses were used to describe the pattern of responses on the MCSDS – Form C. In addition, the tetrachoric correlation matrix between the items was calculated. Tetrachoric correlation is a special case of polychoric correlation specifically used with ordinal dichotomous data (Pearson, 1900; Carrol, 1961), as is the case in the MCSDS – Form C. Furthermore, to test the psychometric performance of the MCSDS – Form C in Kazakhstan, we used a five-step approach that included (1) dimensionality reduction, (2) exploration of factorial structure, (3) confirmation of factorial structure, (4) analysis of measurement invariance across gender, age, language, and geographic location, and (5) factorial and composite reliability analysis (see Figure 1).

The factorial structure of the MCSDS – Form C was first examined using several dimensionality reduction approaches. First, a Principal Component Analysis (PCA) was implemented on the matrix of tetrachoric correlations. The Kaiser criterion, the results of parallel analyses, and the interpretation of the scree plot were used to determine the number of factors underlying the structure of the scale. Second, a Categorical Principal Component Analysis (CATPCA) conducted on the raw data was used to further explore the dimensionality of the scale. CATPCA is a technique of optimal scaling designed specifically for categorical ordinal and nominal data with the ability to account for non-linear relations between variables. Instead of a linear combination of transformed variables, the method transforms, through iterative computation, the matrix of actual categorical data into quantified data with further maximization of eigenvalues on the matrix of quantified data (Gifi, 1990; Linting et al., 2007).

The resulting dimensions were further analyzed using an Exploratory Factor Analysis (EFA) computed on the matrix of tetrachoric correlations. The robust weighted least squares (WLS) estimator was used to account for the dichotomous nature of the scale. The robust version uses only diagonal elements of the weight matrix to obtain standard errors (Muthen et al., 1997), whereas the standard version employs a full weight matrix (Browne, 1984). Both robust and standard estimators are asymptotically free. However, the robust WLS shows stable results in samples of different sizes, while the standard WLS shows stability only in large samples (Flora and Curran, 2004; Barendese et al., 2014).

The resulting factor structures were tested using a Confirmatory Factor Analyses (CFA) correlated factor models with a diagonally weighted least square estimator (DWLS), as suggested by Brown (2006). In addition, we tested alternative, more complex factor structures such as bifactor and hierarchical factor models. The former allows to model separate effects of specific and general factors while the later accounts for the direct effect of the higher order factor on the first order factors. The Chi-square test (χ^2^) was used to evaluate the absolute fit of the model. However, because the χ^2^ test is considered highly conservative, additional fit indices were used to evaluate the model, such as the Comparative Fit Index (CFI), the Tucker-Lewis Index (TLI), and the Root Mean Square Error of Approximation (RMSEA). The values of CFI and TLI > 0.95 and RMSEA < 0.06 indicated a good model fit, while CFI and TLI > 0.90 and RMSEA < 0.08 indicated a satisfactory fit (Hu and Bentler, 1999; Schreiber et al., 2006). Finally, to offer an alternative account of the factorial structure of the scale, we conducted a Random Intercept Item Factor Analysis (RIIFA) to test whether the results of the MCSDS – Form C contain a method factor along with the substantial factor representing social desirability. For instance, this can be due to negatively and positively worded items (Marsh, 1996; DiStefano and Motl, 2009) in survey instruments. The effect of a method factor can be found via modeling residual covariance separately between positive and negative items (Marsh, 1989, 1996) or by allowing intercept in a CFA model to vary across respondents in a Random Intercept Item Factor Analysis (RIIFA, Maydeu-Olivares and Coffman, 2006; Nieto et al., 2021). In the latter, one needs to add one method factor and set its loadings to 1 with free estimated variance. The approach is appropriate to model individual styles of responses and helps to identify whether a multidimensional structure is truly due to substantive factors or due to a spurious, method factor which goes along with the substantive factor. Hence, we run an additional RIIFA model and check the fit statistic and variance of the random component.

Further, we tested configural (unconstrained), metric (constrained slopes), and scalar (constrained slopes and intercepts) measurement invariance across gender, age, language, and geographic location using Multiple Group Confirmatory Factor Analysis (MGCFA). The likelihood ratio test was used to compare statistically significant changes between different models at the *p* < 0.05 level. A non-statistically significant change was interpreted as the indication supporting measurement invariance (Satorra and Bentler, 2000).

Finally, after exploring the dimensionality and testing the measurement invariance of the scale, we examined the factorial and composite reliability of the scores. To investigate the reliability of the Kazakhstani version of the MCSDS – Form C, we calculated the Cronbach alpha coefficient on the matrix of tetrachoric correlations of the full scale. However, when the instrument does not have Tau-Equivalent items (equal factor loadings) and shows multidimensionality, alpha is not the optimal solution. Moreover, the alpha coefficient often serves as a lower bound or largely underestimates reliability (Sijtsma, 2009). Furthermore, when multidimensionality is detected via the CFA framework, a more appropriate alternative is to use the omega reliability coefficient (McDonald, 1999; Green and Yang, 2015; Flora, 2020). Omega calculates reliability of the scale that is due to the presence of some general factor in bifactor and hierarchical models as well as group-specific factors (Green and Yang, 2015). In this study, we focus on composite reliability, or in other words, the sum of factor loadings of individual items. We calculated the ω coefficient for correlated factors in the CFA models and also show the composite alpha coefficient.

All calculations were carried out with the R statistical programming language (R Core Team, 2020). The PCA was performed using the *FactoMiner* package with PCA function (Le et al., 2008). The CATPCA was performed using the *gifi* package and the *princals* function (Mair et al., 2019). EFAs were performed using the *psych* package, with the *fa* function (Revelle, 2021). CFA and measurement invariance tests were calculated using the specialized package for structural equation modeling *lavaan* (Version 0.6-9; Rosseel, 2012). Reliability analysis was calculated with the *SEMTools* package (Version 0.5-5; Jorgensen et al., 2021). The R scripts with all calculations are provided as Supplementary Material.

## Results

### Descriptive Statistics

The response pattern for the MCSDS – Form C items is presented in Table 2. We recalculated socially desirable responses as 1 (socially desirable response is detected) and 0 (no socially desirable response is detected). In the table, the dichotomy is presented in the form of “yes” and “no.” In general, the results suggest high levels of social desirability bias for all items, except items 1 (59.6%) and 2 (49.0%). Table 2 also depicts the matrix of tetrachoric correlations between the items. The correlation ranges from low negative r_tet_ > −0.1 between items 13 and 12 to moderate positive r_tet_ < 0.58 between items 7 and 5. For some pairs of items (e.g., 13 and 2, 13, and 3), the correlation is essentially 0, suggesting the absence of statistical interdependence.

**TABLE 2:** Pattern of responses across items and matric of tetrachoric correlation (*n* = 2,407).

	Yes (%)	No (%)	Item 1	Item 2	Item 3	Item 4	Item 5	Item 6	Item 7	Item 8	Item 9	Item 10	Item 11	Item 12
Item 1	59.6	40.4	–											
Item 2	49.0	51.0	0.50	–										
Item 3	65.8	34.2	0.35	0.42	–									
Item 4	85.6	14.4	0.37	0.41	0.39	–								
Item 5	94.1	5.9	−0.03	−0.03	−0.06	−0.01	–							
Item 6	91.3	8.7	0.30	0.26	0.18	0.37	0.20	–						
Item 7	96.8	3.2	0.03	−0.03	−0.09	0.01	0.58	0.23	–					
Item 8	89.2	10.8	0.14	0.24	0.27	0.33	0.14	0.45	0.17	–				
Item 9	90.6	9.4	0.06	0.12	0.07	0.05	0.50	0.16	0.53	0.26				
Item 10	70.8	29.2	0.05	0.15	0.08	0.06	0.33	0.11	0.40	0.12	0.36	–		
Item 11	84.7	15.3	0.24	0.34	0.33	0.28	0.10	0.40	0.07	0.32	0.06	0.14	–	
Item 12	77.5	22.5	0.30	0.34	0.25	0.34	0.15	0.35	0.17	0.33	0.17	0.11	0.26	–
Item 13	72.8	27.2	−0.01	0.00	0.00	0.03	0.19	0.13	0.26	0.10	0.27	0.45	−0.07	−0.09

### Dimensionality Reduction

**FIGURE 2:**
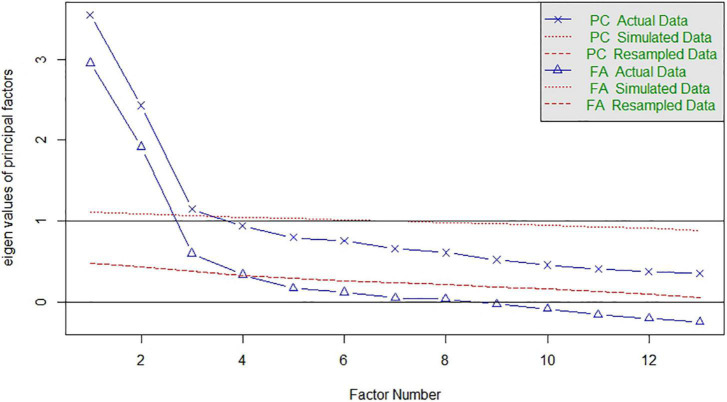
Scree plot and parallel analysis.

Table 3 shows the PCA results on the matrix of tetrachoric correlations for the first five components. The analysis yielded three components with eigenvalues greater than 1, accounting for 72.33% of the total variance. However, the leveling of the eigenvalues on the scree plot and the results of the parallel analysis do not provide a definitive answer to the dimensionality of the scale (see Figure 2).

**TABLE 3:** Results of PCA and CATPCA (*n* = 2.407).

Linear PCA				CATPCA		
Component	Eigenvalue	% of variance explained	Cumulative% of variance explained	Eigenvalue	% of variance explained	Cumulative% of variance explained
1	6.541	50.315	50.315	2.27	17.50	17.50
2	1.807	13.904	64.219	1.67	12.82	30.33
3	1.054	8.114	72.334	1.12	8.61	38.95
4	0.757	5.829	78.163	1.03	7.94	46.89
5	0.724	5.576	83.740	0.89	6.85	53.75

**FIGURE 3:**
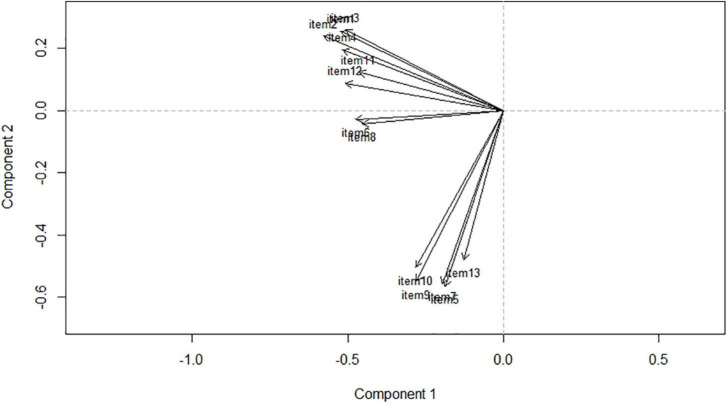
CATPCA loadings plot.

Both the two- and the three-component solutions appear as plausible solutions. Alternatively, we explored the dimensionality of the scale by running CATPCA on the actual data. As in linear PCA, we looked at eigenvalues and the explained variance or variance accounted for (VAF) to understand how many components to retain. Furthermore, eigenvalues larger than 1, as well as the scree plot, can help to decide the adequate number of components (Linting et al., 2007). The results suggest at least two clear dimensions with eigenvalues of 2.27 and 1.67 and a cumulative variance explained of 30.33%. With the inclusion of the third component with an eigenvalue of 1.12, the cumulative variance increases from 30.33 to 38.95%. Figure 3 also suggests at least two clear components with a plausible additional third component. Overall, the results of the dimensionality reduction techniques suggest the existence of two or three components underlying the structure of the MCSDS – Form C.

### Exploration of Factorial Structure

The two- and three-component structures were further examined using EFAs with oblique rotation on the matrices of tetrachoric correlations. The results of the EFA for the two- and three-factorial solutions are presented in Table 4. The two-factor solution demonstrated acceptable loadings (i.e., >0.40) for the 13 items of the MCSDS – Form C. Eight items load on factor 1, which explained 20% of the variance. Five items demonstrated loadings on factor 2, accounting for 17% of the variance. The high uniqueness of item 13 is noteworthy (0.84). In addition, item 6 and item 8 load on both factors, although loadings on factor 1 are at least two times larger than on factor 2. The correlation between the two factors was modest (*r* = 0.22).

**TABLE 4:** Results of the EFAs for the two- and three-factorial solutions (*n* = 2,407).

	Two-factor model				Three-factor model				
	Factor 1	Factor 2	h2	μ2	Factor 1	Factor 2	Factor 3	h2	μ2
Item 1	0.60		0.34	0.66	0.61			0.35	0.65
Item 2	0.68		0.45	0.55	0.71	−0.20		0.48	0.52
Item 3	0.60		0.34	0.66	0.62	−0.22		0.36	0.64
Item 4	0.64		0.40	0.60	0.64			0.40	0.60
Item 5		0.72	0.50	0.50		0.77		0.56	0.44
Item 6	0.52		0.35	0.65	0.49	0.25		0.37	0.63
Item 7		0.79	0.61	0.39		0.76		0.62	0.38
Item 8	0.47		0.29	0.71	0.45	0.22		0.30	0.70
Item 9		0.67	0.47	0.53		0.58	0.20	0.45	0.55
Item 10		0.52	0.29	0.71			0.55	0.47	0.53
Item 11	0.53		0.28	0.72	0.51	0.20		0.29	0.71
Item 12	0.52		0.30	0.70	0.50			0.35	0.65
Item 13		0.41	0.16	0.84			0.66	0.48	0.52

*Factor loadings < 0.20 are omitted.*

The three-factorial solution achieved similarly acceptable item loadings. The same eight items loaded into factor 1. The remaining items loaded into factor 2 (3 items) and factor 3 (2 items). Factors 1, 2, and 3 explained 20, 15, and 7% of the total variance, respectively. Since we allowed factors to correlate, one can notice that items 6, 8, and 10 have additional loadings on factor 2. There was a moderate correlation between factor 1 and factor 2 (*r* = 0.27) and between factor 2 and factor 3 (*r* = 0.26). However, no statistically significant relationship was found between factor 1 and factor 3 (*r* = 0.02).

Overall, the results of the EFAs suggest that these factorial structures could be a result of theoretical dimensions of SDB but also due to methodological influences related to the keyed direction of the items of the scale. In the next section, several factor theoretical and methodological solutions are tested using CFAs.

### Confirmation of Factorial Structure

**FIGURE 4:**
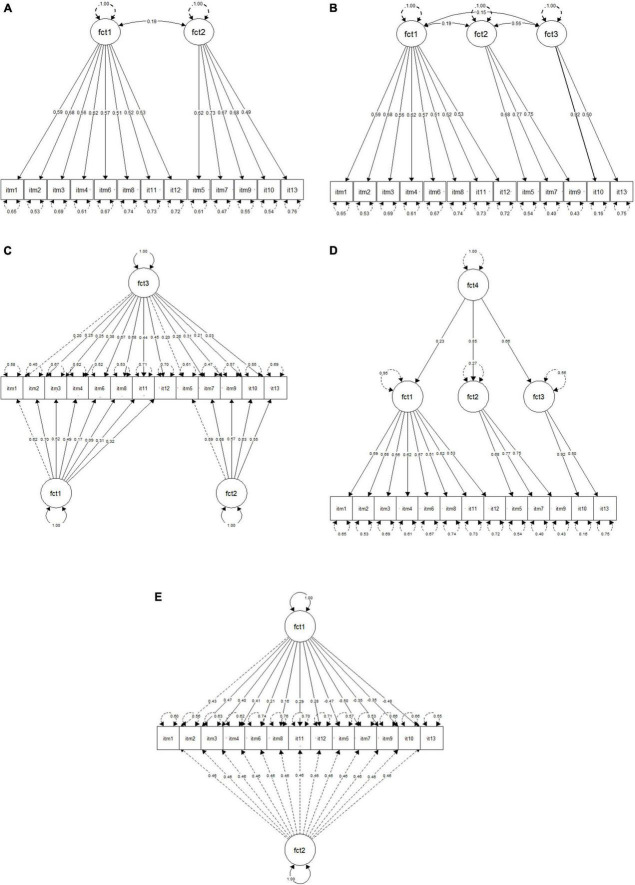
Standardized factor loadings for the two-, three-, bifactor, hierarchical, and random item intercept models of the MCSDS – form C (*n* = 2,407). **(A)** Two-factor model. **(B)** Three-factor model. **(C)** Bifactor model *. **(D)** Hierarchical three-factor model. **(E)** Random item intercept factor model. * Loading between factor 2 and item 5 is fixed to 1 for model identification. Covariances between specific factors and between general and specific factors are fixed to 0.

Confirmatory Factor Analyses were conducted to examine the structural validity of the two-factor and three-factor solutions emerging from the EFA, as well as their more complex alternatives (i.e., bifactor and hierarchical factor models). Furthermore, for reasons of comparison and to test the hypothetical one-factor structure of the MCSDS – Form C, we run a CFA for the unidimensional model. As in the EFA analysis, the parameter estimates in the models were obtained using the robust diagonally weighted least squares (DWLS) estimator to account for the dichotomous nature of MCSDS – Form C. Table 5 presents the robust fit indices of the calculated models. As indicated by the χ^2^ values, none of the models fit perfectly. In line with the multidimensional structure revealed in previous analyses, the unidimensional solution indicated the worst fit. The two-factor model was found to have an absolute satisfactory fit, with standard CFI = 0.94, TLI = 0.93, and RMSEA = 0.035. The three-factor model also achieved a satisfactory fit, with CFI = 0.95, TLI = 0.94, and RMSEA = 0.030. Although both models demonstrated a satisfactory fit, the differences in TLI, CFI, and RMSEA between the two models demonstrated the superiority of the three-factor model. In addition, since we used the DWLS estimator, the difference between the nested models was calculated with a scaled Satorra-Bentler chi-square difference test (Satorra and Bentler, 2000). In support of the comparison between the fit indices, there was a statistically significant difference between the two- and the three-factor models with a *p*-value of 4.958e-07. Figures 4A,B presents the standardized path estimates for both models. All standardized path estimates were significantly loaded into the hypothesized specific factors in the two-factor (β = 0.49 to 0.73, *p* < 0.01) and three-factor models (β = 0.50 to 0.77, *p* < 0.01).

**TABLE 5:** CFA and RIIFA comparison of standard fit statistics (robust is given in parenthesis, *n* = 2,407).

Model	RMSEA	TLI	CFI	χ^2^	degrees of freedom	*p*-value
One-factor model	0.071 (0.073)	0.709 (0.624)	0.757 (0.686)	856 (887)	65	0
Two-factor model	0.035 (0.036)	0.931 (0.905)	0.943 (0.922)	249 (268)	64	0
Three-factor model	0.030 (0.033)	0.947 (0.922)	0.958 (0.938)	198 (224)	62	0
Bifactor model with two specific factors	0.024 (0.029)	0.967 (0.940)	0.978 (0.959)	126 (160)	53	0
Hierarchical model with three first order factors	0.030 (0.033)	0.947 (0.922)	0.958 (0.938)	198 (224)	62	0
Random Intercept One Factor model	0.032 (0.035)	0.940 (0.914)	0.951 (0.929)	225 (248)	64	0

*The bifactor model with three specific factors was tested but failed to be identified. Hierarchical models with two first order factors tend to be underidentified (Brown, 2006) and was therefore not tested in this study.*

The bifactor solution with two specific factors showed the highest TLI = 0.967 and CFI = 0.978 and the lowest RMSEA = 0.024 which indicated the best absolute fit among the calculated models. However, notwithstanding the fit indices, the model had poor loadings (<0.40) between general factor and a set of items, ranging from β = 0.03 to 0.38 (Figure 4C). The bifactor solution with three specific factors failed to be identified. Thus, despite the best absolute fit, the three-factor model can be still regarded as superior to the bifactor solution. We also calculated a hierarchical model with three first order factors and one second order factor. The standard fit statistics of the higher order model produced identical results to the three-factor correlated model. However, it is useful to look at factor estimates as well as loadings between the first and the second order factors (Figure 4D). The results of the standardized solution showed weak loading of higher level with factor 1 (β = 0.23), high but not statistically significant loading with factor 2 (β = 0.85, *z*-value = 1.801), and moderate high with factor 3 (β = 0.66). The rest of the loadings between the second order latent variables and items were essentially identical to the three-factor model. Finally, since hierarchical models with two second order factors are considered to be underidentified (Brown, 2006), we did not try to extract the general factor from the two-factor model.

Models examined above accounted for substantive, theory driven factors. Table 5 presents the fit statistics of the RIIFA model to test the specific variance associated with the item keying as a result of a methodological artifact. Standard fit statistics demonstrated a satisfactory fit for the RIIFA model, with TLI = 0.940, CFI = 0.951, RMSEA = 0.032. In addition, the estimate of random component variance accounted for about 21% of all variances with significant *z* = 23.65 and std. error = 0.009. This is larger than the variance the substantive factor where the estimate is 0.18 with *z* = 6.90 and std. error = 0.027. However, some factor loadings in the RIIFA model were relatively small (β < 0.40) (see Figure 4E).

Based on the findings above, we proceeded to explore measurement invariance for the two and three-factor models. The one-factor model was not further analyzed because of the unsatisfactory fit. Due to low loadings and no statistical significance between latent variables (general and specific) and some observed variables in the bifactor solution as well as first and second order factors in the hierarchical solution, these models were not further tested for measurement invariance and reliability either. Also, the RIIFA was not further explored for measurement invariance and reliability due to the low factor loadings of some of the items (e.g., items 6, 8, 11, and 12).

### Measurement Invariance Across Gender, Age, Language, and Geographic Location

The MGCFA results for measurement invariance for the two- and three-factor solution of the MCSDS – Form C across gender (male vs. female), age (18–35 year old vs. 36–50 year old vs. 51–72 year old), language (Russian vs. Kazakh), and geographic location (urban vs. rural) are presented in Table 6.

**TABLE 6:** Measurement invariance.

	Two-factor model		Three-factor model	
Group	MI	*p* value	MI	*p* value
Rural-urban	Configural – metric	0.51	Configural – metric	0.50
	Metric – scalar	0.43	Metric – scalar	0.74
Age	Configural – metric (partial)	0.08	Configural (failed) – metric	–
	Metric (partial) – scalar	0.11	Metric – scalar (failed)	–
Gender	Configural – metric	0.16	Configural – metric (failed)	–
	Metric – scalar (failed) Configural – scalar	–0.52	Metric (failed) – scalar Configural – scalar	–0.56
Language	Configural – metric	0.46	Configural – metric	0.56
	Metric – scalar (partial)	0.70	Metric – scalar (partial)	0.53

For the two-factor solution, the MGCFA did not show statistical significance and therefore full configural-metric and full metric-scalar invariance for rural and urban teachers with *p* = 0.51 and *p* = 0.43, respectively. The analysis established partial configural-metric invariance (*p* = 0.08) with item 3 being freed up in the constrained loadings model for factor 1 and partial metric-scalar invariance (*p* = 0.11) among teachers from different age groups where in addition to item 3, we allowed loadings between factor 1 and items 4 and 6 to vary between groups. Furthermore, while the analysis did not show statistical significance between the configural and metric models for gender with *p* = 0.16, the invariance between the metric and configural models was not reached (*p* = 0.02). The Lagrange Multiplier Test did not indicate significant items with all *p*-values above the threshold of 0.05. As in the MGCFA analysis for the three-factor solution, the likelihood ratio test between the configural and scalar models did not show statistically significant differences with *p* = 0.52. Finally, for the language group, we found no difference (*p* = 0.46) between the general model with varied intercepts and loadings across Russian and Kazakh speaking teachers and partial invariance (*p* = 0.70) with items 2 and 3 being freed up for factor 1 in the scalar model.

For the three-factor solution, measurement invariance was established between rural and urban participants with *p* = 0.50 between configural – metric and *p* = 0.75 between metric – scalar. The same was true in the Russian-Kazakh language of the questionnaire, with *p* = 0.56 between configural – metric and *p* = 0.53 between metric – scalar. It is important to point out that the scalar model for language showed a statistically significant difference with the metric model and thereby we switched to partial solution freeing up loadings for items 2, 3, and 6 in factor 1. For age, the configural and scalar models failed to demonstrate measurement invariance, as some estimated variances showed negative signs. For gender, we encountered the same problem with metric invariance. However, comparing the configural model with the scalar model, the *p*-value was 0.56.

Overall, these findings demonstrate the measurement invariance of the MCSDS – Form C across language and geographic location for both models, but not across gender groups in the two- and three-factor solutions and age in the three-factor solution.

### Factorial and Composite Reliability

Two approaches were implemented to explore the reliability of the scores in the two models under examination for the Kazakhstani version of the MCSDS – Form C. First, internal consistency was examined using Cronbach’s alpha (α) coefficient. The results demonstrated adequate internal reliability for the two dimensions of the two-factor model (α = 79, α = 76, respectively). For the three-factor model, internal reliability was adequate for factor 1 (α = 79) and factor 2 (α = 77), but lower for factor 3 (α = 62). Second, to account for the multidimensionality of the scale, the reliability of the scores was examined using the McDonald’s omega (ω) statistic. Coefficient ω for subscale internal consistency exhibited poor reliability indices for the two dimensions in the two-factor (ω = 0.54, ω = 0.50, respectively). Similarly, coefficient ω for the three dimensions in the three-factor model were low, ranging from 0.47 to 0.54. We do not specifically discuss an acceptable threshold of reliability in this paper, but we expect group-specific factors to be higher than 0.70 to be counted as at least acceptable.

## Discussion

This research investigated the psychometric performance of the Marlowe-Crowne Social Desirability Scale (MCSDS) – Form C in a nationally representative sample of teachers in Kazakhstan. We examined the factorial structure of the scale using several dimensionality reduction techniques, such as Principal Component Analysis (PCA) and Categorical Principal Component Analysis (CATPCA), as well as Exploratory Factor Analysis (EFA) computed on the matrix of tetrachoric correlations. Furthermore, the theoretical structure of the scale was further tested using a Confirmatory Factor Analysis (CFA) and a Random Intercept Item Factor Analysis (RIIFA). We tested whether the measure varied between gender, age, geographic location, and language groups using Multigroup Confirmatory Factor Analyses (MGCFA). Finally, the reliability of the scores was explored using Cronbach’s alpha and McDonald’s omega coefficients.

Overall, the results of this study do not support the theoretical unidimensionality of the Kazakhstani version of the scale (Reynolds, 1982). In contrast, the findings clearly suggest that a multidimensional factorial structure and existence of a spurious factor provide better representations of the data. On the one hand this is consistent with a growing number of studies that have challenged the use of the full and short versions of the MCSDS to measure a single factor of SDB representing “need for approval” (e.g., Paulhus, 1984; Barger, 2002; Stöber et al., 2002; Leite and Beretvas, 2005). On the other hand, the significant random component along with the substantive component supports the idea that the results of MCSDS-Form C were affected by the response style of the teachers (Maydeu-Olivares and Coffman, 2006).

The results of this study suggest that both a two and a three correlated factor models demonstrated satisfactory fit to the data in the CFAs. Their more complex alternatives (i.e., bifactor and hierarchical factor models) were underidentified or demonstrated low factor loadings for some of the items. Although the three-factor model showed a relatively better performance than the two-factor model, the later seemed to provide a more empirically adequate and theoretically sound structure for the Kazakhstani version of the MCSDS – Form C. This could be due to at least four reasons. First, the EFA with oblique rotation showed substantial item cross-loadings (>0.20) for the three-factor model. Such cross-loadings present a great challenge for classical CFA, since significant cross-loadings can affect model estimation and identification (Mai et al., 2018; Zhang et al., 2021). Second, the moderate to high correlation between the second and third factors (*r* < 0.56) in the three-factor model suggests that both factors essentially represent one construct. Furthermore, the low correlation between the two components in the two-factor and three-factor CFA models (*r* < 0.20) suggests that these two are separate but related constructs. Third, the test of measurement invariance across age and language in the three-factor model showed improper solution and non-convergence issues. This can be due to the small number of indicators (i.e., two items) for factor 3. Such results are in line with findings on estimation and convergence in CFA models. For instance, Anderson and Gerbing (1984) found that the likelihood of non-convergent and improper cases increases in models with small sample sizes and a small number of indicators per factor. Similarly, Ding et al. (1995) showed that the frequency of improper solutions depends on small samples and two indicators per factor in CFA models. For the two-factor model, we did not have non-convergence and improper solutions across all groups, although we found statistical differences between men and women teachers. Fourth, the internal consistency coefficients demonstrate slightly better reliability of the scores in the two-factor solution compared to the three-factor solution. More specifically, the alpha coefficients suggest that the items of the scale are relatively accurate when measuring two dimensions, but they do not precisely measure a third dimension (α = 0.62). However, the low omega coefficients for all subscale scores (ω < 0.60) indicate that neither the two-factor nor the three-factor models offer high confidence in measuring SDB with an acceptable level of precision.

**FIGURE 5:**
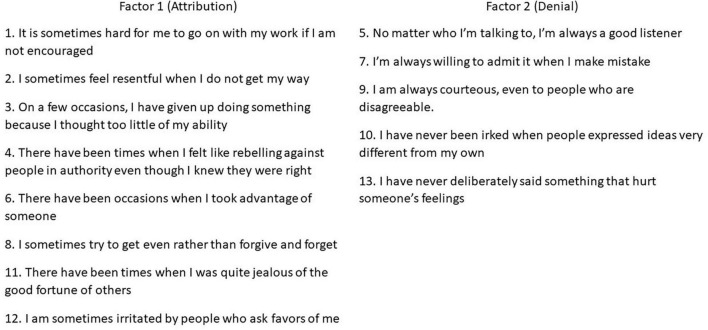
Distribution of items across the two latent factors in the Kazakhstani MCSDS – Form C.

In addition to these reasons, the two-factor model also presents itself as a better solution from a theoretical point of view. Figure 5 presents the resulting distribution of items across the two latent factors. The Kazakhstani version of the MCSDS Form C seems to resemble two separate dimensions of social desirability: attribution and denial (Millham, 1974). The former accounts for assigning socially favorable traits to oneself, while the latter represents a tendency to deny socially unfavorable traits. Furthermore, existing studies of the original MCSDB scale over the years in different cultural contexts confirmed that attribution and denial are the two underlying dimensions of the full as well as the short forms (Ramanaiah et al., 1977; Loo and Thorpe, 2000; Tao et al., 2009; He et al., 2015; Kurz et al., 2016). In this context, it can be argued that the first factor accounts for the dimension of attribution, whereas the second factor represents the dimension of denial. Individuals with high scores on both constructs, rather than being concerned with the actual meaning of their behavior, are more concerned with the external disapproving judgment (Millham, 1974). Furthermore, based on the low factor correlation (*r* < 0.20) we support the idea that these two sub concepts should be measured separately (Fischer and Fick, 1993).

Alternatively, the RIIFA model demonstrated the existence of a spurious factor associated with the item keying. In this model, the random component accounted for the substantial percentage of variance (21%), whereas the substantial factor accounted for 18%. The bigger proportion of variance of the random intercept suggests that the scale answers depend more on the method factor rather than the substantial factor that represents SDB. Thus, unlike the two-factor solution, the second dimension is not substantive and merely depicts idiosyncratic use of the scale by the teachers. Moreover, in comparison with the two-factor solution, the RIIFA model produced a relatively better fit. Overall, in this particular sample of Kazakhstani teachers, these findings present an alternative interpretation of the MCSDS-Form C results that do not support the existence of the attribution and denial dimensions. Moreover, the RIIFA results indicate low factor loadings between the substantial factor and items 6, 8, 11, 12 (β = 0.16, β = 0.29) suggesting weak relation between the items and the substantive factor, as well as the clear grouping of negatively and positively worded items.

Collectively, based on the results above, we favor the RIIFA solution and suggest interpreting the results of MCSDS-Form C as dependent on teacher response styles, not on the substantive factors representing social desirability. Still, the two-factor solution can be considered as a good hypothetical alternative that should be considered when working with MCSDS-Form C.

This is especially relevant considering some striking results in the latest TALIS 2018 study. For instance, in Kazakhstan 72% of teachers self-assessed their level of preparedness in classroom management as good and very good. In comparison, the OECD average in this component was 53% (OECD, 2019). In fact, in all items on preparedness Kazakhstani teachers indicated higher percentages of good and very good levels than their colleagues from OECD, the range of percentage difference is from 9 to 22% (Information-Analytic Center [IAC], 2019).

A plausible explanation for the high percentages of SDS in the present study is the higher number of females in the sample. In fact, the population distribution indicates a proportion of 4 to 1 (80 to 20%) in favor of female teachers (Information-Analytic Center [IAC], 2020). Previous research has shown that females tend to exhibit higher SDS than male respondents (e.g., Barger, 2002; Booth-Kewley et al., 2007; Fastame and Penna, 2012; Bossuyt and Van Kenhove, 2018). Apart from this, some broader cultural differences, such as collectivism and individualism, may lead to differences in responses. High levels of SDB in collectivist societies (e.g., like Kazakhstan) have been widely discussed in the literature (Middleton and Jones, 2000; van Hemert et al., 2002; Kim and Kim, 2016; Ryan et al., 2021). For example, van Hemert et al. (2002) found a negative correlation between the Lie scale and individualist culture. The Lie scale constitutes a part of EPQ (Eysenck Personality Questionnaire) and measures social conformity and behavior of faking good (Eysenck and Eysenck, 1991). Thus, one of the possible major reasons behind the poor reliability of the MCSDB – Form C in this study could relate to the general tendency to give dishonest answers according to collectivist cultural orientations in Kazakhstan. Unfortunately, we do not have enough evidence to further elaborate on this point since our primary interest was to check psychometric properties of the short form. Surprisingly, this article is one of the few attempts to study an instrument measuring SDB in a post-soviet country of Central Asia with collectivist culture, even though the social desirability was extensively studied cross-culturally elsewhere, across different fields of social science including but not limited to psychology, education, and sociology. Moreover, a large part of the previous research utilizing full and short forms of MCSDS was mainly concerned with social desirability as representing substantive dimensions but did not consider the potential effect of a response style on the scale answers. In this respect, when working with MCSDS forms we propose to account for both, substantive, and method factors by using traditional CFA and the RIIFA models. More research is needed in this direction. We believe that this article will open a path to future research on social desirability bias as a response pattern and as a personality characteristic with special focus on collectivist post-soviet countries of Central Asia.

Speaking about the limitations of the article, we can highlight several major factors that can potentially affect the results. First, according to the results, the scale is not a perfect measurement of social desirability; ideally, it would be appropriate to repeat the above procedure on the full MCSDB scale consisting of 33 items. This article focuses only on one of the existing short forms proposed by Nederhof (1985). The second limitation is related to the target population of the survey and its subgroups’ specifics. Although the sample is representative, it focuses only on the subject teachers. Sampling issues are not new or specific to this particular Kazakhstani MCSD survey. Many studies have identified sampling representations as limitations (Beretvas et al., 2002; Sârbescu et al., 2012). Although some of these studies indicate an overwhelming participation of males (Sârbescu et al., 2012), other studies find issues of reliability differences on social desirability even with less differences in gender representation (Loo and Thorpe, 2000; Beretvas et al., 2002). Thus, future research on SDB in Kazakhstan and in societies with predominantly collectivist culture can broaden the focus from specific target subpopulations to the general country-wide population testing either several short forms or the full MCSDB scale. Third, although the MCSDB scale is one of the most widely spread instruments, there are other traditional scales (Edwards, 1957; Sackheim and Gur, 1978; Paulhus, 1988; Eysenck and Eysenck, 1991) that can be used together with the MCSDB to measure social desirability and to test for convergent validity. The fourth limitation concerns measurement invariance for the RIIFA model. Although due to low factor loadings we did not calculate configural, metric and scalar invariance models nevertheless future research could include traditional MI as well as computation of a specific (factor and method) metric invariance to test whether the substantive factor and the method factor are independent (Steenkamp and Maydeu-Olivares, 2020).

In addition, factor analysis works best with the continuous data, employed in this study on the matrix of tetrachoric correlation, it is a limited information model, and the results must be regarded as an approximation of the full model (Mislevy, 1986; Schumacker and Beyerlein, 2000). Therefore, in exploring the factorial structure of MCSDS – Form C, future research can focus on full information models that allow one to work directly with categorical data and account for potentially important cross-loadings. Instead of the classical approach used in this article, one could use either Bayesian CFA or MIRT models. In the former, one can account for important cross-loadings in the model by placing normal priors with small variance on them (Muthen and Asparouhov, 2012). In the latter, MIRT models specifically work with categorical binary and polytomous items and allow estimation of within item structure where an item can be associated with several latent traits, which is not possible in classical CFA.

## Conclusion

Research on SDB requires measurement instruments that provide reliable and valid scores in local contexts, cultures, and languages. In this study, we report several approaches to determine the psychometric performance of the Kazakhstani version of the MCSDS – Form C. We conclude that when using the Kazakhstani version of the MCSDS – Form C, if the RIIFA modes does not signal the presence of a significant method factor along with the substantive factor, then separate attribution and denial scores should be used instead of a total score measuring SDB. Furthermore, caution should be exercised when interpreting these scores due to the low omega reliability coefficients obtained for both subscales. The measurement of attribution and denial is equivalent across geographic location (urban vs. rural), language (Kazakh vs. Russian), and age groups, but these dimensions seem to be interpreted differently between male and female participants. Furthermore, MCSDS does not seem to be a perfect instrument for the context of Kazakhstani teachers because the collective culture of the Kazakhstani society combined with the current rigid vertical system of education could have an impact on the answers to the questions of the instrument. Despite these limitations, the validation of the Kazakhstani version of the MCSDS – Form C presented in this study is a first step in facilitating further research and measurement of SDB in post-Soviet Kazakhstan and other Central Asian countries.

## Data Availability Statement

The datasets presented in this article are not readily available because of organizational data confidentiality policy. Requests to access the datasets should be directed to KN.

## Ethics Statement

Ethical review and approval was not required for the study on human participants in accordance with the Local Legislation and Institutional Requirements. The patients/participants provided their written informed consent to participate in this study.

## Author Contributions

KN and DH-T contributed to the conception and design of the study, organized the database, performed the statistical analysis, and wrote the first draft of the manuscript. AA and UO wrote the sections of the manuscript. All authors contributed to the article and approved the submitted version.

## Conflict of Interest

The authors declare that the research was conducted in the absence of any commercial or financial relationships that could be construed as a potential conflict of interest.

## Publisher’s Note

All claims expressed in this article are solely those of the authors and do not necessarily represent those of their affiliated organizations, or those of the publisher, the editors and the reviewers. Any product that may be evaluated in this article, or claim that may be made by its manufacturer, is not guaranteed or endorsed by the publisher.
