# Supplementary material for: Measuring Social Desirability in Collectivist Countries: A Psychometric Study in a Representative Sample From Kazakhstan
Source: Front Psychol. 2022 Apr 6;13:822931. doi: 10.3389/fpsyg.2022.822931 (PMC9020785; doi:10.3389/fpsyg.2022.822931)
Supplement: Supplementary file 1 [file Data_Sheet_1.pdf]

```
### neccessary libraries
library(openxlsx)
library(psych)
library(lavaan)
library(psych)
library(GPArotation)
library(semPlot)
library(semTools)
library(FactoMineR)
library(reshape)
library(Gifi)
setwd("")# define working directory
df<-read.xlsx('mcsds.xlsx') # data
df<- rename(df, c(X.PERS1='item1', X.PERS2='item2', X.PERS3='item3', X.PERS4='item4',
          X.PERS5='item5', X.PERS6='item6', X.PERS7='item7', X.PERS8='item8',
          X.PERS9='item9', X.PERS10='item10', X.PERS11='item11', X.PERS12='item12',
          X.PERS13='item13'))




### Tetrachoric correlation
tetr.cor.mat<-tetrachoric(df,y=NULL,correct=.5,smooth=TRUE,global=TRUE,weight=NULL,na.rm=TRUE,
           delete=TRUE)
tetr.cor.mat<-tetr.cor.mat$rho


######################## PCA
fit_pca<-PCA(tetr.cor.mat)
fit_pca
########################
```

```
################# fa parallel
fit.fa<-fa.parallel(tetr.cor.mat,n.obs=2407,fm="wls",fa="both",nfactors=3,
        main="Parallel Analysis Scree Plots",
        n.iter=20,error.bars=FALSE,se.bars=FALSE,SMC=FALSE,ylabel=NULL,show.legend=TRUE,
        sim=TRUE,quant=.95,cor="tet",use="pairwise",plot=TRUE,correct=.5)
fit.fa
plot(fit.fa, main=NULL)
###########################


######## categorical principal component
cpca<-princals(df, 3)
plot(cpca, main=NULL)
summary(cpca) # summary of the results
###############################


#################### EFA with two and three dimensions (factors)
fit_efa.two<-fa(tetr.cor.mat, n.obs = nrow(df), 2, fm='wls', rotate = "promax")
fit_efa.two
summary(fit_efa.two)


fit_efa.three<-fa(tetr.cor.mat, n.obs = nrow(df), 3, fm='wls', rotate = "promax")
fit_efa.three
summary(fit_efa.three)


### recode to ordinal (if needed)
df3$item1<-factor(df3$item1, ordered = T, levels = c("1", "0"))
```

```
df3$item2<-factor(df3$item2, ordered = T, levels = c("1", "0"))
df3$item3<-factor(df3$item3, ordered = T, levels = c("1", "0"))
df3$item4<-factor(df3$item4, ordered = T, levels = c("1", "0"))
df3$item5<-factor(df3$item5, ordered = T, levels = c("1", "0"))
df3$item6<-factor(df3$item6, ordered = T, levels = c("1", "0"))
df3$item7<-factor(df3$item7, ordered = T, levels = c("1", "0"))
df3$item8<-factor(df3$item8, ordered = T, levels = c("1", "0"))
df3$item9<-factor(df3$item9, ordered = T, levels = c("1", "0"))
df3$item10<-factor(df3$item10, ordered = T, levels = c("1", "0"))
df3$item11<-factor(df3$item11, ordered = T, levels = c("1", "0"))
df3$item12<-factor(df3$item12, ordered = T, levels = c("1", "0"))
df3$item13<-factor(df3$item13, ordered = T, levels = c("1", "0"))
############

# Model 1 - two dimensions
# Model 2 - three dimensions

Model1 <- '
factor1=~item1+item2+item3+item4+item6+item8+item11+item12
factor2=~item5+item7+item9+item10+item13
factor1~~factor2'

Model2 <- '
factor1=~item1+item2+item3+item4+item6+item8+item11+item12
factor2=~item5+item7+item9
factor3=~item10+item13
factor1~~factor2
factor2~~factor3'


fit.two <- cfa(Model1,data=df, std.lv=TRUE, ordered = c("item1", "item2", "item3", "item4", "item5",
                        "item6",  "item7", "item8", "item9", "item10",
```

```
                                   "item11", "item12", 'item13'))
fit.three <- cfa(Model2,data=df, std.lv=TRUE, ordered = c("item1", "item2", "item3", "item4", "item5",
                                     "item6", "item7", "item8", "item9", "item10",
                                     "item11", "item12", 'item13'))
anova(fit.two, fit.three)
summary(fit.two, fit.measures=TRUE, standardized = TRUE)
summary(fit.three, fit.measures=TRUE, standardized = TRUE)


### sem plots
semPaths(fit.two, what='std', edge.label.cex = 0.7, edge.color = 1, esize=2, sizeMan=6, asize=2.5, intercepts = F,
    thresholdColor = 'red', thresholdSize = 0.01, fade=F, nCharNodes = 4)
semPaths(fit.three, what='std', edge.label.cex = 0.7, edge.color = 1, esize=2, sizeMan=6, asize=2.5, intercepts = F,
    thresholdColor = 'red', thresholdSize = 0.01, fade=F, nCharNodes = 4)
################################
################################
################################
################################
################################




########################### MI across language, gender, age, area (rural-urban) with three factors model


rm(list=ls())
df<-read.xlsx('mcsds_mi.xlsx') # data set with subgroups used for MI
df<- rename(df, c(X.PERS1='item1', X.PERS2='item2', X.PERS3='item3', X.PERS4='item4',
        X.PERS5='item5', X.PERS6='item6', X.PERS7='item7', X.PERS8='item8',
        X.PERS9='item9', X.PERS10='item10', X.PERS11='item11', X.PERS12='item12',
        X.PERS13='item13'))
```

```
######### three factors MGCFA
Model <- '
factor1=~item1+item2+item3+item4+item6+item8+item11+item12
factor2=~item5+item7+item9
factor3=~item10+item13
factor1~~factor2
factor2~~factor3'


#### measurement invariance - configural
cfa.config.area <- cfa(Model,data=df, std.lv=TRUE, group='rural_urban',
                  ordered = c("item1", "item2", "item3", "item4", "item5",
                            "item6", "item7", "item8", "item9", "item10",
                            "item11", "item12", 'item13'))
#summary(cfa.config.area, fit.measures = TRUE, standardized = TRUE)

cfa.config.age <- cfa(Model,data=df, std.lv=TRUE, group='age_group_new',
                 ordered = c("item1", "item2", "item3", "item4", "item5",
                           "item6", "item7", "item8", "item9", "item10",
                           "item11", "item12", 'item13'))
#summary(cfa.config.age, fit.measures = TRUE, standardized = TRUE)

cfa.config.lang <- cfa(Model,data=df, std.lv=TRUE, group='submisson_lang',
                  ordered = c("item1", "item2", "item3", "item4", "item5",
                            "item6", "item7", "item8", "item9", "item10",
                            "item11", "item12", 'item13'))
#summary(cfa.config.lang, fit.measures = TRUE, standardized = TRUE)


cfa.config.gender<-cfa(Model,data=df, std.lv=TRUE, group='gender',
```

```
                    ordered = c("item1", "item2", "item3", "item4", "item5",
                               "item6", "item7", "item8", "item9", "item10",
                               "item11", "item12", 'item13'))
#summary(cfa.config.gender, fit.measures = TRUE, standardized = TRUE)
####################
################ for the age groups some estimated variances are negative




################## metric
cfa.metric.area <- cfa(Model,data=df, std.lv=TRUE, group='rural_urban',
                    ordered = c("item1", "item2", "item3", "item4", "item5",
                               "item6", "item7", "item8", "item9", "item10",
                               "item11", "item12", 'item13'),
                    group.equal = "loadings")
#summary(cfa.metric.area, fit.measures = TRUE, standardized = TRUE)

cfa.metric.age <- cfa(Model,data=df, std.lv=TRUE, group='age_group_new',
                   ordered = c("item1", "item2", "item3", "item4", "item5",
                              "item6", "item7", "item8", "item9", "item10",
                              "item11", "item12", 'item13'),
                   group.equal = ("loadings"))
#summary(cfa.metric.age, fit.measures = TRUE, standardized = TRUE)

cfa.metric.lang <- cfa(Model,data=df, std.lv=TRUE, group='submisson_lang',
                    ordered = c("item1", "item2", "item3", "item4", "item5",
                               "item6", "item7", "item8", "item9", "item10",
                               "item11", "item12", 'item13'),
                    group.equal = "loadings")
#summary(cfa.metric.lang, fit.measures = TRUE, standardized = TRUE)
```

```
cfa.metric.gender<- cfa(Model,data=df, std.lv=TRUE, group='gender',
                    ordered = c("item1", "item2", "item3", "item4", "item5",
                               "item6", "item7", "item8", "item9", "item10",
                               "item11", "item12", 'item13'),
                    group.equal = "loadings")

########### for the gender, some estimated variances are negative
#######################




anova(cfa.metric.area, cfa.config.area) # established
anova(cfa.metric.age , cfa.config.age) ## configural model contains estimated negative variances
anova(cfa.metric.lang, cfa.config.lang) # established
anova(cfa.metric.gender, cfa.config.gender) ### metric model contains estimated negative variances



############################################ scalar
cfa.scalar.area <- cfa(Model,data=df, std.lv=TRUE, group='rural_urban',
                    ordered = c("item1", "item2", "item3", "item4", "item5",
                               "item6", "item7", "item8", "item9", "item10",
                               "item11", "item12", 'item13'),
                    group.equal = c("loadings", 'intercepts'))
#summary(cfa.scalar.area, fit.measures = TRUE, standardized = TRUE)

cfa.scalar.age <- cfa(Model,data=df, std.lv=TRUE, group='age_group_new',
                   ordered = c("item1", "item2", "item3", "item4", "item5",
                              "item6", "item7", "item8", "item9", "item10",
                              "item11", "item12", 'item13'),
                   group.equal = c("loadings", 'intercepts'))
#summary(cfa.scalar.age, fit.measures = TRUE, standardized = TRUE)
# some estimated variances for the age groups are negative
```

```
cfa.scalar.lang <- cfa(Model,data=df, std.lv=TRUE, group='submisson_lang',
                    ordered = c("item1", "item2", "item3", "item4", "item5",
                              "item6", "item7", "item8", "item9", "item10",
                              "item11", "item12", 'item13'),
                    group.equal = c("loadings", 'intercepts'))
#summary(cfa.scalar.area, fit.measures = TRUE, standardized = TRUE)

cfa.scalar.gender <- cfa(Model,data=df, std.lv=TRUE, group='gender',
                    ordered = c("item1", "item2", "item3", "item4", "item5",
                              "item6", "item7", "item8", "item9", "item10",
                              "item11", "item12", 'item13'),
                    group.equal = c("loadings", 'intercepts'))

#summary(cfa.scalar.area, fit.measures = TRUE, standardized = TRUE)
anova(cfa.metric.area, cfa.scalar.area) # established MI
anova(cfa.metric.age , cfa.scalar.age) ### scalar model for the age groups contains negative estimated variances
anova(cfa.metric.lang, cfa.scalar.lang) # not established needs a partial solution
anova(cfa.metric.gender, cfa.scalar.gender) # not established

anova(cfa.config.gender, cfa.scalar.gender) # established

##################################### partial invariance
lavTestScore(cfa.scalar.lang)
cfatable<-parTable(cfa.scalar.lang)
# .p2. ==  .p76. = factor1=~item2
# .p3. ==  .p77.= factor1=~item3
# .p5. ==  .p79. = factor1=~item6
# .p8. == .p82. = factor1=~item12
# .p18. ==  .p92 = item2 | t1
```

```
# .p19. ==  .p93.= item3  | t1
# .p21. ==  .p95.= item6  | t1
# .p24. ==  .p98.= item12 | t1


cfa.scalar.lang.adj <- cfa(Model, data = df, group = "submisson_lang",
                    ordered = c("item1", "item2", "item3", "item4", "item5",
                            "item6", "item7", "item8", "item9", "item10",
                            "item11", "item12", 'item13'),
                    group.equal = c("loadings", 'intercepts'),
                    group.partial = c("factor1=~item2", 'factor1=~item3', 'factor1=~item6'))
anova(cfa.metric.lang, cfa.scalar.lang.adj) # established partial MI


lavTestScore(cfa.metric.gender)
cfatable<-parTable(cfa.metric.gender)
# .p2. == .p76 = factor1=~item2
#  .p8. == .p82.= factor1=~item12


cfa.metric.gender.adj<-cfa(Model, data = df, group = "gender",
                      ordered = c("item1", "item2", "item3", "item4", "item5",
                              "item6", "item7", "item8", "item9", "item10",
                              "item11", "item12", 'item13'),
                      group.equal = c("loadings"),
                      group.partial = c("factor1=~item2", 'factor1=~item12'))
anova(cfa.metric.gender, cfa.scalar.gender.adj)
#### after the adjustment the estimated model still contains negative estimated variances


########################### MI across language, gender, age, geographic area with two factors model
```

```
rm(list=ls())
df<-read.xlsx('mcsds_mi.xlsx') # data set with subgroups used for MI
df<- rename(df, c(X.PERS1='item1', X.PERS2='item2', X.PERS3='item3', X.PERS4='item4',
               X.PERS5='item5', X.PERS6='item6', X.PERS7='item7', X.PERS8='item8',
               X.PERS9='item9', X.PERS10='item10', X.PERS11='item11', X.PERS12='item12',
               X.PERS13='item13'))

######### two factors MGCFA
Model <- '
factor1=~item1+item2+item3+item4+item6+item8+item11+item12
factor2=~item5+item7+item9+item10+item13
factor1~~factor2'
##############
#### measurement invariance - configural
cfa.config.area <- cfa(Model,data=df, std.lv=TRUE, group='rural_urban',
                   ordered = c("item1", "item2", "item3", "item4", "item5",
                             "item6", "item7", "item8", "item9", "item10",
                             "item11", "item12", 'item13'))
#summary(cfa.config.area, fit.measures = TRUE, standardized = TRUE)

cfa.config.age <- cfa(Model,data=df, std.lv=TRUE, group='age_group_new',
                  ordered = c("item1", "item2", "item3", "item4", "item5",
                            "item6", "item7", "item8", "item9", "item10",
                            "item11", "item12", 'item13'))
#summary(cfa.config.age, fit.measures = TRUE, standardized = TRUE)

cfa.config.lang <- cfa(Model,data=df, std.lv=TRUE, group='submisson_lang',
                   ordered = c("item1", "item2", "item3", "item4", "item5",
                             "item6", "item7", "item8", "item9", "item10",
                             "item11", "item12", 'item13'))
#summary(cfa.config.lang, fit.measures = TRUE, standardized = TRUE)
```

```
cfa.config.gender<-cfa(Model,data=df, std.lv=TRUE, group='gender',
                    ordered = c("item1", "item2", "item3", "item4", "item5",
                               "item6", "item7", "item8", "item9", "item10",
                               "item11", "item12", 'item13'))
#summary(cfa.config.gender, fit.measures = TRUE, standardized = TRUE)
###################



################## metric
cfa.metric.area <- cfa(Model,data=df, std.lv=TRUE, group='rural_urban',
                    ordered = c("item1", "item2", "item3", "item4", "item5",
                               "item6", "item7", "item8", "item9", "item10",
                               "item11", "item12", 'item13'),
                    group.equal = "loadings")
#summary(cfa.metric.area, fit.measures = TRUE, standardized = TRUE)

cfa.metric.age <- cfa(Model,data=df, std.lv=TRUE, group='age_group_new',
                   ordered = c("item1", "item2", "item3", "item4", "item5",
                              "item6", "item7", "item8", "item9", "item10",
                              "item11", "item12", 'item13'),
                   group.equal = ("loadings"))
#summary(cfa.metric.age, fit.measures = TRUE, standardized = TRUE)

cfa.metric.lang <- cfa(Model,data=df, std.lv=TRUE, group='submisson_lang',
                    ordered = c("item1", "item2", "item3", "item4", "item5",
                               "item6", "item7", "item8", "item9", "item10",
                               "item11", "item12", 'item13'),
                    group.equal = "loadings")
```

```
#summary(cfa.metric.lang, fit.measures = TRUE, standardized = TRUE)

cfa.metric.gender<- cfa(Model,data=df, std.lv=TRUE, group='gender',
                    ordered = c("item1", "item2", "item3", "item4", "item5",
                              "item6", "item7", "item8", "item9", "item10",
                              "item11", "item12", 'item13'),
                    group.equal = "loadings")

anova(cfa.metric.area, cfa.config.area) # established
anova(cfa.metric.age , cfa.config.age) ## not established, needs a partial solution
anova(cfa.metric.lang, cfa.config.lang) # established
anova(cfa.metric.gender, cfa.config.gender) ### established

##################### partial solution for the age groups
lavTestScore(cfa.metric.age)
cfatable<-parTable(cfa.metric.age)
##########################################################

# .p3. == .p143.  8.378  1  0.004 facto1~~item3
#  .p4. == .p144.  4.723  1   0.030 factor1=~item4
#  .p5. == .p145.  5.807  1   0.016 factor1=~item6
#  .p7. == .p147.  7.232  1   0.007 factor1=~item11
# .p8. == .p148. 12.894  1  0.000 factor1=~item12

cfa.metric.age.adj <- cfa(Model,data=df, std.lv=TRUE, group='age_group_new',
                     ordered = c("item1", "item2", "item3", "item4", "item5",
```

```
                    "item6", "item7", "item8", "item9", "item10",
                    "item11", "item12", 'item13'),
              group.equal = ("loadings"),
              group.partial = c("factor1=~item3"))


anova(cfa.metric.age.adj , cfa.config.age) # established the partial MI




############################################ scalar
cfa.scalar.area <- cfa(Model,data=df, std.lv=TRUE, group='rural_urban',
             ordered = c("item1", "item2", "item3", "item4", "item5",
                    "item6", "item7", "item8", "item9", "item10",
                    "item11", "item12", 'item13'),
             group.equal = c("loadings", 'intercepts'))
#summary(cfa.scalar.area, fit.measures = TRUE, standardized = TRUE)

cfa.scalar.age <- cfa(Model,data=df, std.lv=TRUE, group='age_group_new',
            ordered = c("item1", "item2", "item3", "item4", "item5",
                   "item6", "item7", "item8", "item9", "item10",
                   "item11", "item12", 'item13'),
            group.equal = c("loadings", 'intercepts'))
#summary(cfa.scalar.age, fit.measures = TRUE, standardized = TRUE)

cfa.scalar.lang <- cfa(Model,data=df, std.lv=TRUE, group='submisson_lang',
             ordered = c("item1", "item2", "item3", "item4", "item5",
                    "item6", "item7", "item8", "item9", "item10",
                    "item11", "item12", 'item13'),
             group.equal = c("loadings", 'intercepts'))
#summary(cfa.scalar.area, fit.measures = TRUE, standardized = TRUE)
```

```
cfa.scalar.gender <- cfa(Model,data=df, std.lv=TRUE, group='gender',
                    ordered = c("item1", "item2", "item3", "item4", "item5",
                              "item6", "item7", "item8", "item9", "item10",
                              "item11", "item12", 'item13'),
                    group.equal = c("loadings", 'intercepts'))

#summary(cfa.scalar.area, fit.measures = TRUE, standardized = TRUE)
anova(cfa.metric.area, cfa.scalar.area) # established MI
anova(cfa.metric.age.adj , cfa.scalar.age) ### need to compare the partial metric model
anova(cfa.metric.lang, cfa.scalar.lang) # not established - needs a partial solution
anova(cfa.metric.gender, cfa.scalar.gender) ## not established  - needs a partial solution


anova(cfa.scalar.gender, cfa.config.gender)


cfa.metric.age.adj2 <- cfa(Model,data=df, std.lv=TRUE, group='age_group_new',
                     ordered = c("item1", "item2", "item3", "item4", "item5",
                               "item6", "item7", "item8", "item9", "item10",
                               "item11", "item12", 'item13'),
                     group.equal = ("loadings"),
                     group.partial = c("factor1=~item3", 'factor1=~item4', 'factor1=~item6'))
anova(cfa.metric.age.adj2 , cfa.scalar.age)  # established the partial MI


##################### partial solution for the lang. group
lavTestScore(cfa.scalar.lang)
cfatable<-parTable(cfa.scalar.lang)
############################################################

#  .p2. == .p72. 21.943  1   0.000 factor1=~item2
```

```
#  .p3. == .p73. 55.340  1   0.000 factor1=~item3

#  .p5. == .p75. 13.218  1  0.000 factor1=~item6

#  .p8. == .p78.  6.368  1  0.012 factor1=~item12



cfa.scalar.lang.adj <- cfa(Model,data=df, std.lv=TRUE, group='submisson_lang',
                           ordered = c("item1", "item2", "item3", "item4", "item5",
                                       "item6", "item7", "item8", "item9", "item10",
                                       "item11", "item12", 'item13'),
                           group.equal = c("loadings", 'intercepts'),
                           group.partial=c('factor1=~item2',  'factor1=~item3'))

anova(cfa.metric.lang, cfa.scalar.lang.adj) # established the partial MI



##################### partial solution for the gender group
lavTestScore(cfa.scalar.gender)
cfatable<-parTable(cfa.scalar.gender)
######################################## all coef-s are non-significant
```
