# Supplementary material for: Measuring Social Desirability in Collectivist Countries: A Psychometric Study in a Representative Sample From Kazakhstan
Source: Front Psychol. 2022 Apr 6;13:822931. doi: 10.3389/fpsyg.2022.822931 (PMC9020785; doi:10.3389/fpsyg.2022.822931)
Supplement: Supplementary file 2 [file Data_Sheet_2.pdf]

```
#rm(list=ls())
library(openxlsx)
library(lavaan)
library(semPlot)
library(semTools)
library(reshape)

setwd("")# define working directory
df<-read.xlsx('') # data
df<- rename(df, c(X.PERS1='item1', X.PERS2='item2', X.PERS3='item3', X.PERS4='item4',
        X.PERS5='item5', X.PERS6='item6', X.PERS7='item7', X.PERS8='item8',
        X.PERS9='item9', X.PERS10='item10', X.PERS11='item11', X.PERS12='item12',
        X.PERS13='item13'))




### recode to ordinal (if needed)
df$item1<-factor(df$item1, ordered = T, levels = c("1", "0"))
df$item2<-factor(df$item2, ordered = T, levels = c("1", "0"))
df$item3<-factor(df$item3, ordered = T, levels = c("1", "0"))
df$item4<-factor(df$item4, ordered = T, levels = c("1", "0"))
df$item5<-factor(df$item5, ordered = T, levels = c("1", "0"))
df$item6<-factor(df$item6, ordered = T, levels = c("1", "0"))
df$item7<-factor(df$item7, ordered = T, levels = c("1", "0"))
df$item8<-factor(df$item8, ordered = T, levels = c("1", "0"))
df$item9<-factor(df$item9, ordered = T, levels = c("1", "0"))
df$item10<-factor(df$item10, ordered = T, levels = c("1", "0"))
df$item11<-factor(df$item11, ordered = T, levels = c("1", "0"))
df$item12<-factor(df$item12, ordered = T, levels = c("1", "0"))
df$item13<-factor(df$item13, ordered = T, levels = c("1", "0"))
```

```
############

#### random intercept
Model.riifa <- '
factor1=~item1+item2+item3+item4+item6+item8+item11+item12+item5+item7+item9+item10+item13
factor2=~1*item1+1*item2+1*item3+1*item4+1*item5+1*item6+1*item7+1*item8+1*item9+1*item10+1*item11+1*item12+1*item13
factor1~~0*factor2'
fit.riifa <- cfa(Model.riifa, data=df, ordered = c("item1", "item2", "item3", "item4", "item5",
                                                   "item6",   "item7", "item8", "item9", "item10",
                                                   "item11", "item12", 'item13'))
summary(fit.riifa, fit.measures=TRUE, standardized = T)
#anova(fit.one, fit.two)
semPaths(fit.riifa, what='std',  edge.label.cex = 0.7, edge.color = 1, esize=2, sizeMan=6, asize=2.5, intercepts = F,
         thresholdColor = 'red', thresholdSize = 0.01, fade=F, nCharNodes = 4, rotation=1, edge.label.position=0.7, bifactor='factor1',
         layout='tree2')

## variance of the random component is 0.214 (with s.e. - 0.009)

# bifactor model
Model_bi <- '
factor1=~item1+item2+item3+item4+item6+item8+item11+item12
factor2=~item5+item7+item9+item10+item13
```

```
factor3=~item1+item2+item3+item4+item6+item8+item11+item12+1*item5+item7+item9+item10+ite
m13

'

fit.bi <- cfa(Model_bi, data=df, ordered = c("item1", "item2", "item3", "item4", "item5",

                                             "item6",   "item7", "item8", "item9", "item10",

                                             "item11", "item12", 'item13'), orthogonal = TRUE)

summary(fit.bi, fit.measures=TRUE, standardized = T)

semPaths(fit.bi, what='std', edge.label.cex = 0.7, edge.color = 1, esize=2, sizeMan=6, asize=2.5,
intercepts = F,

        thresholdColor = 'red', thresholdSize = 0.01, fade=F, nCharNodes = 4, bifactor='factor3',
layout='tree2')

#####

###### setting one loading to 1 solves identification

###




## second order factor model with three first order factors

Model_hi <- '

factor1=~item1+item2+item3+item4+item6+item8+item11+item12

factor2=~item5+item7+item9

factor3=~item10+item13

factor4=~factor1+factor2+factor3'

fit.hi <- cfa(Model_hi, data=df, std.lv=T, ordered = c("item1", "item2", "item3", "item4", "item5",

                                             "item6",   "item7", "item8", "item9", "item10",

                                             "item11", "item12", 'item13'))

summary(fit.hi, fit.measures=TRUE, standardized = T)

semPaths(fit.hi, what='std', edge.label.cex = 0.7, edge.color = 1, esize=2, sizeMan=6, asize=2.5,
intercepts = F,

        thresholdColor = 'red', thresholdSize = 0.01, fade=F, nCharNodes = 4)
```

###
####################
